# Effects of Acute Sepsis on Cellular Dynamics and Amyloid Formation in a Mouse Model of Alzheimer’s Disease

**DOI:** 10.3390/cimb44090262

**Published:** 2022-08-24

**Authors:** Alexandra Daniela Rotaru-Zavaleanu, Alexandru Ionuț Neacșu, Adela-Daria Neacșu, Daniel Pirici, Eugen Osiac, Bogdan Cătălin, Dan Ionuț Gheonea

**Affiliations:** 1 Department of Gastroenterology, University of Medicine and Pharmacy of Craiova, 200349 Craiova, Romania; 2 Experimental Research Center for Normal and Pathological Aging, Department of Functional Sciences, University of Medicine and Pharmacy of Craiova, 200349 Craiova, Romania; 3 Department of Biophysics, University of Medicine and Pharmacy of Craiova, 200349 Craiova, Romania; 4 Department of Histology, University of Medicine and Pharmacy of Craiova, 200349 Craiova, Romania; 5 Department of Physiology, University of Medicine and Pharmacy of Craiova, 200349 Craiova, Romania

**Keywords:** sepsis, amyloid deposits, cortical inflammation, bacterial peritonitis, neuronal death

## Abstract

Our objective was to investigate how sepsis influences cellular dynamics and amyloid formation before and after plaque formation. As such, APP-mice were subjected to a polymicrobial abdominal infection resulting in sepsis at 2 (EarlySepsis) and 4 (LateSepsis) months of age. Behavior was tested before sepsis and at 5 months of age. We could not detect any short-term memory or exploration behavior alterations in APP-mice that were subjected to Early or LateSepsis. Immunohistochemical analysis revealed a lower area of NeuN^+^ and Iba1^+^ signal in the cortex of Late compared with EarlySepsis animals (*p* = 0.016 and *p* = 0.01), with an increased astrogliosis in LateSepsis animals compared with WT-Sepsis (*p* = 0.0028), EarlySepsis (*p* = 0.0032) and the APP-Sham animals (*p* = 0.048). LateSepsis animals had larger areas of amyloid compared with both EarlySepsis (*p* = 0.0018) and APP-Sham animals (*p* = 0.0024). Regardless of the analyzed markers, we were not able to detect any cellular difference at the hippocampal level between groups. We were able to detect an increased inflammatory response around hippocampal plaques in LateSepsis compared with APP-Sham animals (*p* = 0.0003) and a decrease of AQP4 signal far from Sma^+^ vessels. We were able to show experimentally that an acute sepsis event before the onset of plaque formation has a minimal effect; however, it could have a major impact after its onset.

## 1. Introduction

One of the most severe life-threatening conditions treated by intensive-care physicians is sepsis [1]. As the result of a complex and exaggerated inflammatory response of the host to infection [2], sepsis represents an acute medical emergency, but due to a plethora of medical strategies, in-hospital mortality rates caused by sepsis have decreased from around 27.8% (in 1979–1984) to 17.9% (in 1995–2000) [3]. However, this improvement also means that surviving patients require a longer rehabilitation program. This is mostly attributed to the fact that up to 71% of septic patients can develop acute cerebral dysfunction [4]. Although not directly involved in the pathology, the brain can be affected by a series of alterations ranging from ischemic lesions and microglia activation to neurotransmitter and blood–brain barrier (BBB) dysfunction. While long-term clinical consequences of most pathological mechanisms are difficult to assess, in the case of sepsis, the disruption of the BBB is associated with a specific pathology called septic encephalopathy (SAE). As such, investigating the potential role of the AQP4 channel in abdominal sepsis seems essential to better understanding clinical recovery as AQP4 is a key player in brain edema [5].

Cortical alterations secondary to sepsis can be observed clinically as a reduced mnesic functions, impaired attention, disorientation, delirium, or even coma [6,7]. Follow-up studies of sepsis survivors have reported long-lasting impairment of memory and executive functions [8], resulting in a drop in patients’ quality of life [9]. This data is also supported by experimental studies reporting that animals subjected to sepsis suffer long-lasting difficulties in performing behavioral tasks [10]. The main theory explaining the observed outcomes identifies neurodegeneration triggered by systemic inflammation as the aggravating factor [11]. With the increase in life expectancy, the chance that an individual suffering from a chronic disease will also have an acute life-threatening event are becoming higher. With the peak risk of developing sepsis being 50 years of age [12] and up to 13.8% of individuals between the ages of 75 and 84 years affected by dementia [13], the theoretical number of patients suffering from both diseases at the same time, is around 60,000 individuals per year in the EU alone [14]. Although recent preclinical studies suggest that systemic inflammation in sepsis may affect the progression of AD [8] and that inflammation can induce or intensify brain dysfunction [15], we found no data reporting on AD patients that suffered an acute sepsis event in their early life before the onset of the disease.

With evidence that pathological changes in AD, specifically amyloid beta deposition, occur up to three decades earlier than the onset of symptoms [16], the way multiple pathologies impact one another is becoming a more concerning issue in modern medicine. As such, in the present study, we decided to evaluate the neuropathological changes induced by sepsis in an animal model of AD in order to assess if sepsis increases the amyloid burden. We thus investigated one of the most important mechanisms driving Abeta (Aβ) deposition (i.e., microglia uptake and perivascular drainage mediated by AQP4). Therefore, our study targets Presenilin and APP mutation carriers who develop early onset AD pathology and relies on the fact that respiratory tract infections in bedridden patients are frequently the cause of death in AD.

## 2. Materials and Methods

### 2.1. Animals

**Figure 1:**
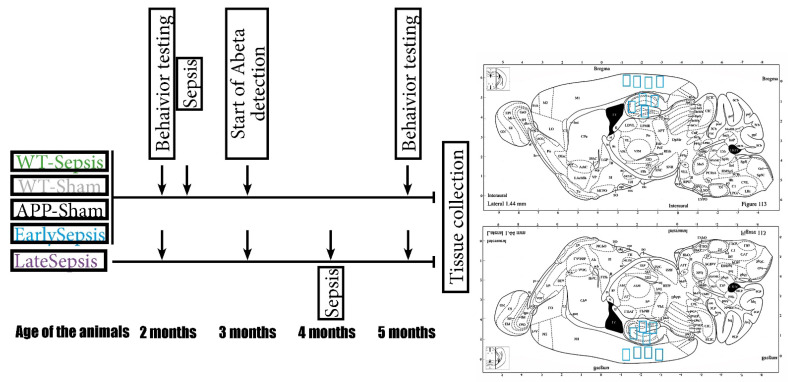
Schematic study design. All animals were tested before the start of any experimental procedure. The WT-Sepsis, APP-Sham and EarlySepsis groups were subjected to CLP at 2 months of age. At 4 months of age, the animals assigned to LateSepsis were subjected to CLP. Behavior testing was performed at 5 months of age for all animals. Tissue collection and consecutive histology analysis was done at 5 months of age for all animals. Diagram showing regions collected for histological analysis (blue squares). For each mouse, the mean of all collected regions of interest was used in the final analysis.

For this study, C57BL/6J-TgN (Thy1-APPKM670/671NL; Thy1-PS1L166P (APP)) mice or wild types were used [17,18]. The mice were bred and housed (in individual ventilated cages under standard room temperature, humidity and day/night cycle) in the Animal Facility of the University of Medicine and Pharmacy of Craiova. All animals used in this experiment were obtained from five mating cages (harem with one male and three females per cage). The APP mice, which are reported to develop plaques at around 3 months of age, were randomly assigned into different groups: EarlySepsis (*n* = 6, 2 months of age at the time of abdominal sepsis), LateSepsis (*n* = 8, 4 months of age at the time of abdominal sepsis), APP-Sham (*n* = 4, 2 months of age at the time of abdominal sham surgery), WT-Sepsis (*n* = 4, 2 months of age at the time of abdominal sepsis) and WT-Sham (*n* = 4, 2 months of age at the time of abdominal sham surgery) animals (Figure 1).

The animals were moved to the testing rooms at least 24 h before the experiment, where they were kept until the end of the study. Before sepsis induction, all animals underwent behavioral testing (as described below). All procedures performed were in accordance with Directive 2010/63/EU of the European Parliament and the Council, approved by the University Welfare of Experimental Animals committee (2.12/29.10.2020) and renewed by the Veterinary Sanitary and Food Safety Directorates of Dolj County (14/29.03.2022), according to Romanian and European laws.

### 2.2. Mouse Model of Cecal Ligation and Puncture

The cecal ligature and puncture model (CLP) was used to induce sepsis [19,20] and early signs of brain dysfunction [21,22]. Before any surgical procedure, deep anesthesia was induced using an intraperitoneal (i.p.) mix of Ketamine (120 mg/kg) and Xylazine (12 mg/kg). Any surgical procedure started only after the animals had no pinch reflex present. CLP surgery was performed according to already described methods [22]. Briefly, animals were placed on the operating table with their abdomen exposed. A longitudinal skin incision was made to expose the peritoneal cavity via the linea alba. After we identified and isolated the cecum it was ligated at 1 cm from the end, paying attention not to also ligate the ileocecal valve. The ligated cecum was then punctured using a 20 G needle, from one side to another, and a small amount of feces was then extruded from the punctures after removing the needle. After the cecum was reinserted into the peritoneal cavity the abdomen was sutured. To mimic the hyperdynamic phase of sepsis, we then administered 1 mL of preheated saline solution, under the skin, with the mouse in the anatomical position. After surgery, the animal was placed alone in the cage, in close vicinity to a heating source and was monitored for the next 12 h. In order to easily verify signs of sepsis, a modified murine sepsis scale was used, as previously described [22,23]. In short, appearance, consciousness, activity, response to stimulus, opening and closing of the eyes and respiration were evaluated. Additionally, intrarectal temperature was recorded every 4 h.

### 2.3. Behavioral Testing

Behavior testing was performed blinded on all mice at 2 months of age, before sepsis surgery and at 5 months of age prior to perfusion. To reduce the interference between tests, open filed (OF) was performed first followed by the novel object recognition (NOR) test. All surfaces were wiped with 75% ethanol before each trial to remove odors.

For the OF test, mice were placed in an open field maze (50 × 33 × 15 cm). At the beginning, all animals were positioned in the center of the arena and were allowed to move and explore the arena for 10 min. The animal behavior was automatically analyzed (EthoVision XT 14, Noldus Technology, Wageningen, The Netherlands) and several parameters (time spent in the center arena, traveled distance and speed) were determined. One animal was not interested in exploring the arena and was excluded from OF quantifications.

The NOR test was used to assess short-term memory. The animals were placed in an arena along with two identical objects situated at 15 cm from the side walls in two opposite parts of the maze. Every animal had 6 min to explore the maze. After 60 min, the animals were placed again in the same arena, but one of the objects was replaced with a new one. The tested mouse was allowed to explore the maze with the new object for 6 min. For each animal, the discrimination index (D2) [24] (the percentage of the time spent exploring the novel object compared with the total time spent exploring both objects) was determined.

Tissue sampling was performed at 5 months of age. The animals were placed under deep anesthesia using a mix of Ketamine (120 mg/kg) and Xylazine (12 mg/kg) administered i.p. An intracardiac perfusion with saline solution (to remove the blood from the circulatory system) followed by formaldehyde for fixation (to prevent unwanted microglia activation) were performed [25]. The brain was removed and kept in formaldehyde for 24 h. After this interval, the tissue was placed in paraffin blocks for histological analysis.

### 2.4. Double Immunohistochemistry

For the immunohistochemistry (IHC) analysis, seriate sections were cut, deparaffinized, re-hydrated, and first processed for antigen retrieval in citrate buffer (0.1 M, pH 6) by microwaving them for 20 min at 650 W. After cooling down to room temperature, endogenous peroxidase was inhibited with a 1% solution of water peroxide for 30 min, then the unspecific binding sites were blocked in 3% skimmed milk (Biorad, California, CA, USA) for another 30 min. The slides were next incubated simultaneously with different pairs of primary antibodies as follows: rabbit anti-GFAP (1:20,000, Dako, Glostrup, Denmark)/mouse anti-NeuN (1:500, Merck Millipore, Temecula, CA, USA), rabbit anti-iba1 (1:1000, Thermo Fisher Scientific, Waltham, MA, USA)/mouse anti-Abeta (4G8 clone, 1:20,000, Signet, Dedham, MA, USA), and rabbit anti-AQP4 (1:1000, Santa Cruz Biotechnology, Dallas, TX, USA)/mouse anti-SMA (1:50 Dako) for 18 h at 4 °C. The following day, the signal was amplified, sequentially, with a goat anti-rabbit alkaline-phosphatase conjugated polymer (Vector Laboratories, Burlingame, CA, USA) for 1 h and then with a goat anti-mouse peroxidase-conjugated polymer for another 1 h (Vector Laboratories, Burlingame, CA, USA). After thorough washing, the signals were detected sequentially, first with Fast Red substrate for alkaline phosphatase (Vector Laboratories), and then with 3,3′-diaminobenzidine (DAB) (Vector Laboratories). After a Hematoxylin counterstaining, the slides were coverslipped with a glycerol-based mounting (Dako). Images were collected randomly from the regions of interest (cortex and hippocampus), capturing three microscopic fields (671.38 × 562.95 µm) for each region in sections cut 1 to 2 mm lateral of the sagittal suture.

### 2.5. Data Acquisition and Statistical Analysis

Using a Zeiss Axio (Zeiss, Oberkochen, Germany) scanner microscope, cortical and hippocampal images (335.69 × 281.48 µm – 2452 × 2056 pixels) were captured. The area of NeuN, GFAP, Iba1, Aβ and AQP4 signals was determined using ImagePro Plus 7 software (Media Cybernetics Corporation, Rockville, MD, USA), with automated and constant image segmentation performed utilizing a user defined RGB spectral signature of the DAB signal. The average cortical and hippocampal signal area was determined for each mouse and for each immunostaining. For each mouse, between 4 and 8 regions of interest were acquired for the cortex and for the hippocampus. The average signal area was used for each mouse in the final analysis. To assess AQP4 and Iba1 around the arteries and plaques, a mask was created allowing for signal area evaluation only at the investigated distance from the SMA of Aβ. The average area determined for each animal was then processed using Prism 9 (GraphPad Software, San Diego, California, Redmond, Washington, DC, USA) and Excel (Microsoft, Redmond, WA, USA). For statistical analysis, ANOVA (Tukey Multiple Comparation Test) was used to ensure more power in multiple compilations. The number of animals used allows for at least 90% power for all histological findings. The figures presented are for individual animal value, mean value and standard deviation (SD). If not stated otherwise, statistical significance is indicated as follows * *p* < 0.05, ** *p* < 0.01, *** *p* < 0.001, **** *p* < 0.0001.

## 3. Results

### 3.1. Severity of Sepsis Is Similar in All Animals Used

**Figure 2:**
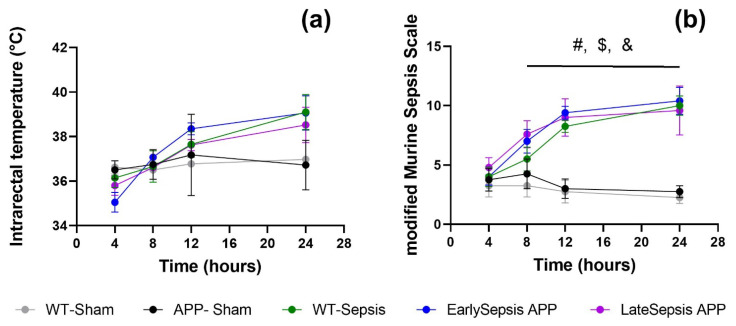
Systemic response to sepsis within the first 24 h: (**a**) animals that were subjected to the CLP had increased intrarectal temperatures compared with APP-Sham animals regardless of the group that they were assigned to; (**b**) the overall status of the animals worsens with all animals subjected to CLP having higher severity scores compared with sham, but with no difference between them. The graphs show mean values ± SD. # APP-Sham-WT-Sepsis, $ APP Sham-EarlySepsis, & APP Sham-LateSepsis.

Evaluating the severity of sepsis allowed for homogenous cellular and behavior results. The monitoring of intrarectal temperature and the use of a modified Murine Sepsis Scale (mMSS) [22] allowed for a minimal invasive evaluation of the overall wellbeing of the animals. Four hours after the surgery, the core temperature of the animals dropped to around 35.87 ± 0.61 °C (Figure 2a). At 24 h the animals subjected to sepsis experienced mild fever with a core temperature around 38.35 ± 1.11 °C. At the same 24 h mark, mMMs reached the highest score 10 ± 0.6 (Figure 2b), showing that the overall wellbeing of the animal had started to be affected.

### 3.2. Sepsis Does Not Alter Short Term Memory or Exploration Behavior of APP Mice

**Figure 3:**
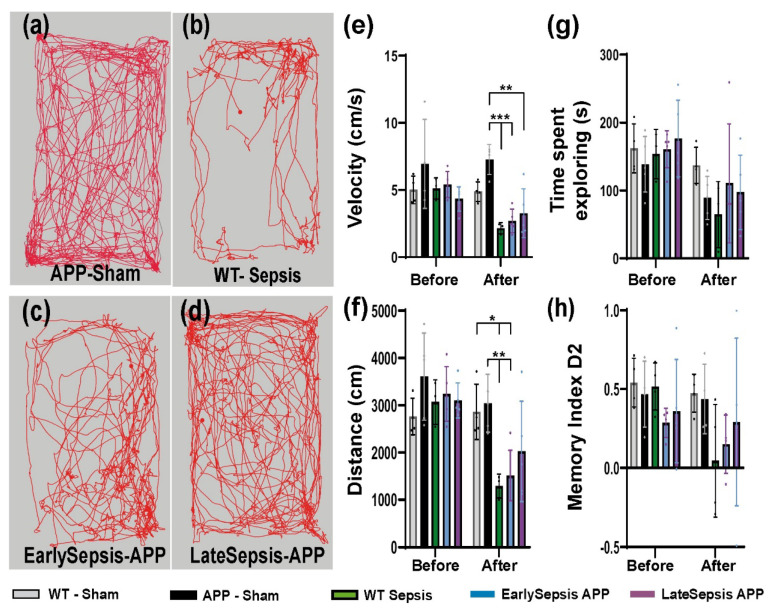
Behavior testing in APP mice subjected to sepsis. Open field examples of (**a**) App-Sham, (**b**) WT animals subjected to sepsis, (**c**) EarlySepsis and (**d**) LateSepsis performed after sepsis. Testing reveals that (**e**) animals subjected to CLP were slower in their exploration (2.24 ± 0.38 cm/min for WT-Sepsis, 2.87 ± 0.87 cm/min for EarlySepsis and 3.2 ± 1.63 cm/min for LateSepsis) (*p* < 0.0001) compared with animals that were not subjected to sepsis (7.27 ± 1.11 cm/min in APP Sham). (**f**) EarlySepsis and WT-Sepsis animals explored shorter distances of 1512.13 ± 534.03 cm (*p* = 0.0049) and 1241.17228.81 cm (*p* = 0.0016), respectively, compared with APP-Sham (3048.52 ± 706.48 cm). (**g**) LateSepsis animals did not display this behavior, exploring an average distance of 2039.57 ± 951 cm (*p* > 0.05). (**h**) Regardless of group, all animals showed similar exploration times. The graphs show mean values ± SD, * *p* < 0.05, ** *p* < 0.01 and *** *p* < 0.001.

Testing performed before and after CLP showed that some behavior changes appeared in animals subjected to sepsis regardless of the present of amyloid deposits. Specifically, animals that were subjected to abdominal sepsis were slower in their exploration (2.24 ± 0.38 cm/min for WT-Sepsis, 2.87 ± 0.87 cm/min for EarlySepsis and 3.2 ± 1.63 cm/min for LateSepsis) (*p* < 0.0001) (Figure 3e) compared with animals that were not subjected to sepsis (7.27 ± 1.11 cm/min in APP Sham animals). EarlySepsis and WT-Sepsis animals explored shorter distances of 1512.13 ± 534.03 cm (*p* = 0.0049) and 1241.17 ± 228.81 cm (*p* = 0.0016), respectively, compared with APP-Sham animals (3048.52 ± 706.48 cm) (Figure 3f). LateSepsis animals did not display this behavior, exploring an average distance of 2039.57 ± 951 cm (*p* > 0.05). Regardless of the group, all animals showed similar exploration time (Supplementary Table S1) (Figure 3g).

When testing the impact that sepsis has on the memory of APP mice, we observed that all animals performed similarly in the NOR test before and after the acute sepsis event, regardless of their status, *p* > 0.05 (Supplementary Table S1) (Figure 3h).

### 3.3. Sepsis Impacts the Cortex and Hippocampus Differently

**Figure 4:**
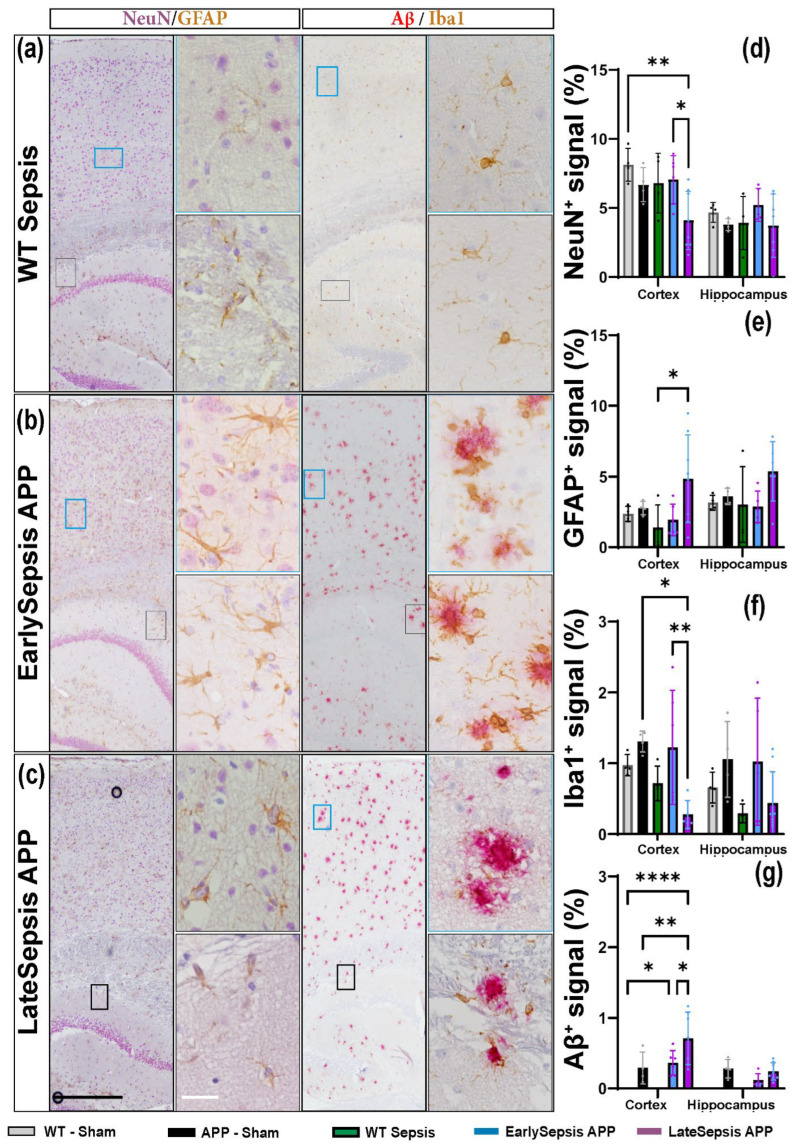
APP and WT mice brain cellular response to sepsis. (**a**–**c**) Double staining of cortex and hippocampus shows that (**d**) the area of NeuN^+^ signal in the cortex of LateSepsis animals was lower than in EarlySepsis animals (4.09 ± 2.11 vs. 7.04 ± 1.73%; *p* = 0.016). (**e**) Increased astrogliosis was observed in LateSepsis (4.84 ± 3.10%) animals compared with WT-Sepsis (1.30 ± 1.60%, *p* = 0.0028), EarlySepsis (1.94 ± 1.11%, *p* = 0.0032) and APP-Sham animals (2.77 ± 0.47%, *p* = 0.048). (**f**) The area of cortical Iba1^+^ signal was higher in the EarlySepsis group (1.22 ± 0.80%) compared with the LateSepsis group (0.28 ± 0.20%, *p* = 0.01). (**g**) LateSepsis animals had larger areas of amyloid (0.71 ± 0.37%) compared with both EarlySepsis animals (0.36 ± 0.17%, *p* = 0.0018) and APP-Sham animals (0.29 ± 0.24%, *p* = 0.0024). Regardless of the analyzed markers, we were not able to detect any difference at the hippocampal level between groups. Black scale bar: 100 µm, White scale bar: 10 µm. The graphs show mean values SD, * *p* < 0.05, ** *p* < 0.01, ****, *p* < 0.0001.

We tested the cellular impact of sepsis in both the cortex and hippocampus. We only observed changes at the cortical level, with no changes within the hippocampus (Figure 4). The area of NeuN^+^ signal in the cortex of LateSepsis animals (4.09 ± 2.11%) was lower than in EarlySepsis animals (7.04 ± 1.73%, *p* = 0.016) and WT-Sham mice (8.14 ± 1.19%, *p* = 0.0045). No difference was observed when comparing the NeuN^+^ signal of APP-Sham mice (6.69 ± 1.25%) and WT-Sepsis animals (6.80 ± 2.16%, *p* > 0.05) (Figure 4d). Cortical astrogliosis was observed in the LateSepsis animals (4.84 ± 3.10 %) compared with WT-Sepsis animals (6.80 ± 2.16%, *p* = 0.0028), EarlySepsis animals (1.94 ± 1.11%, *p* = 0.0032), APP-Sham animals (2.77 ± 0.47%, *p* = 0.048) (Figure 4e). In order to investigate inflammation-elicited sepsis on the brain, we quantified the Iba1 signal. The cortical Iba1^+^ signal was higher (1.22 ± 0.80%) in the EarlySepsis group compared with the LateSepsis group (0.28 ± 0.20%; *p* = 0.01) (Figure 4f). We were not able to identify any difference in Iba1^+^ signal between EarlySepsis and LateSepsis animals (*p* = 0.056) (Supplementary Table S2).

In order to quantify the Aβ changes, we analyzed plaque areas in 5-month animals, regardless of CPL timing. We found larger areas of amyloid (0.71 ± 0.37%) in LateSepsis animals compared with both EarlySepsis animals (0.36 ± 0.17%, *p* = 0.0018) and APP-Sham animals (0.29± 0.23%, *p* = 0.0024) (Figure 4g). Regardless of the signal analyzed, we were not able to detect any difference at the hippocampal level (Supplementary Table S2).

**Figure 5:**
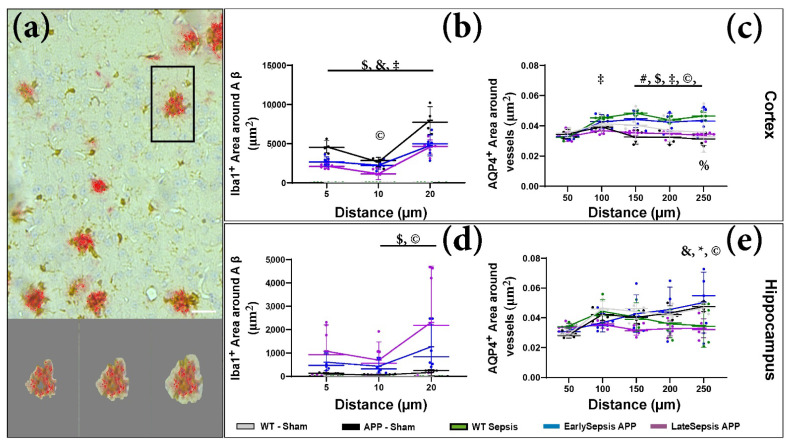
Signal quantification around target. (**a**) After the mask was selected, we determined the signal area within a certain distance from the target. (**b**) Analysis of the Iba1 signal around Aβ positive sites did show any cortical difference in the inflammatory response between APP-Sham and any APP mice subjected to sepsis. (**c**) Quantification of cortical AQP4 distribution around Sma^+^ vessels in APP and WT mice subjected to sepsis. Up to 250 µm there were large differences between groups (Supplementary Table S3), especially between EarlySepsis and LateSepsis mice. (**d**) The only difference found at the hippocampal level was that LateSepsis animals had an increase in the area of Iba1^+^ signal 20 µm from the plaque (2309.53 ± 2412.18 µm^2^) compared with APP-Sham animals (163.94 ± 127.07 µm^2^, *p* = 0.0003). (**e**) The same analysis at the hippocampal level revealed a different pattern of distribution compared with that seen in the cortex. Scale bar: 20 µm. The graphs show mean values, SD and significance between different groups represented by % WT-Sham-WT-Sepsis, # APP-Sham-WT-Sepsis, $ APP Sham-EarlySepsis, & APP Sham-LateSepsis, * WT-Sepsis-EarlySepsis, ‡ WT-Sepsis-LateSepsis, © EarlySepsis-LateSepsis.

To closer evaluate Aβ induced inflammation we quantified the amount of Iba1^+^ signal around the plaques (Figure 5a). The analysis did not show any cortical difference in the inflammatory response between APP-Sham and Early or LateSepsis animals (Figure 5b). The only difference found at the hippocampal level was that LateSepsis animals had an increase in the area of Iba1^+^ signal 20 µm from the plaque (2309.53 ± 2412.18 µm^2^) compared with App-Sham animals (163.94 ± 127.07 µm^2^, *p* = 0.0003) (Figure 5d).

We know that AQP4 distribution around arteries (Sma^+^ vessels) can change in aging [26,27] or when the BBB is altered [28]. As such, we wanted to identify any difference in the AQP4 distribution around Sma^+^ after sepsis. By determining the average AQP4^+^ signal within concentric areas around the vessels (Figure 5c), we were able to identify a difference between AQP4 distribution in EarlySepsis and LateSepsis mice (Supplementary Table S3). The dynamics of AQP4 distribution seems to also differ in the cortex and hippocampus, with more AQP4 distribution around cortical Sma^+^ vessels between 150 µm (0.043 ± 0.004 positive signal/µm^2^ vs. 0.035 ± 0.002 positive signal/µm^2^, *p* = 0.0048) and 250 µm (0.041 ± 0.008 positive signal/µm^2^ vs. 0.034 ± 0.002 positive signal/µm^2^, *p* = 0.0059) in EarlySepsis compared with LateSepsis mice (Figure 5c). However, no difference was observed between the AQP4 cortical distribution between APP-Sham and LateSepsis (Supplementary Table S3). At the hippocampal level, the distribution of AQP4 around Sma^+^ vessels was different between Late and EarlySepsis mice only at the edge of the analyzed field, with EarlySepsis mice displaying more AQP4 positive signal/µm2 (0.054 ± 0.006 µm^2^) compared with LateSepsis mice (0.033 ± 0.006 µm^2^), (*p* = 0.0033) (Figure 5e).

## 4. Discussion

With life expectancy increasing, the chance of an acute life-threatening event affecting an individual suffering from a chronic disease is also increasing. In 2018, 10,935,444 cases of dementia were reported in Europe (EU28 + 9 others countries) [29]. Within the same interval around 3,400,000 people suffered a sepsis episode [30]. This means, at least theoretically, it was possible that approximately 60,000 Europeans with dementia also suffered from sepsis in 2018. This number is purely theoretical, as sepsis affects younger individuals. However, this raises a different issue, as just by chance some adults that had sepsis could also develop AD. This by itself, will not pose a large problem, but patients that develop sepsis are also frequently affected by one of the most underrated types of encephalopathies in the form of SAE [31,32]. SAE can be described as a diffuse cerebral disfunction that appears as a response to the body’s inflammatory response, without directly affecting the CNS [33,34]. SAE, and by extension sepsis, is such a complex pathology, its long-term consequences can be unpredictable, ranging from depression and cognitive effects to a plethora memory impairments [4]. In recent years, the concept of SAE and its effects during an acute episode of sepsis have been studied in detail, both in human and animal models. However, the exact long-term effects are still unknown. This is primarily because most patients with a history of a septic episode are difficult to follow long term.

The onset of AD neurodegenerative processes initially occurs at subclinical level and is manifested by the presence of amyloid plaques [16]. The plaque formation process can occur up to three decades before the clinical onset of symptoms, and therefore, long before the actual diagnosis of AD [35,36]. The most common findings in Alzheimer disease are: the presence of amyloid plaques, neuroinflammation and neuronal death [37]. The age of onset of these subclinical lesions is around 65 years. With sepsis being more likely to induce sepsis-related organ lesions in the elderly due to lower natural defenses [38], the clinical approach in such cases seems to be even more complex than we are now imagining it.

Here, we wanted to establish a cellular baseline for comparison between the groups. As such, we analyzed the number of neurons (NeuN^+^ cells), astrocytes (GFAP^+^ cells) and inflammatory cells (Iba1^+^) present in the cortex and hippocampus of the tested animals. By analyzing these histological signals, we quantified all the major pathological aspects reported in AD patients, from cellular processes such as neuronal loss [39] and neuroinflammation, astrocytic proliferation, and increased expression of the intermediate filaments during glial inflammation [40] to molecular processes such as the altered AQP4 distribution around arteries vessels [28].

Recent experimental data linked severe episodes of sepsis to a faster progression of amyloid plaques development, as well as a worsening of clinical manifestations [18]. Experimental models of sepsis can mimic different aspects of sepsis, with CLP evoking similar inflammatory response to that following a polymicrobial abdominal infection so that from a pathophysiological point of view, it generates similar conditions found in bacterial peritonitis in humans [17]. The inflammatory response in CLP is sustained due to polymicrobial infection, so that from a pathophysiological point of view, it best mimics the conditions of bacterial peritonitis in humans [41]. By applying the CLP sepsis model to APP/PS1 (a transgenic mouse model of AD in which Aβ can be detected as early as 6 weeks of age in the cortex and at 8 weeks of age in the hippocampus [42]) we were able to simulate a sepsis episode before (EarlySepsis group) and after (LateSepsis group) the start of Aβ deposits in the CNS of the animals. It had been established that body temperature variation is one of the most important predictors of survivability in CLP models [23], making the monitoring of temperature vital for our experiment. However, one of the most interesting practical aspects of mice abdominal surgery was the observation that body temperature varies more in a midline abdominal incision [43]. By also including an APP-Sham group, we ensured that any variations induced by changes in body temperature and the midline abdominal incision were taken in account [23].

The CPL model evoked similar clinical manifestations in the animals used, with similar central temperature increases (Figure 1), ensuring a similar cortical response. With several studies reporting changes in mice exploratory activity and anxiety-related behaviors after sepsis [22,44], we investigated how sepsis impacted the behavior of APP mice. Locomotion (“total distance moved” and “velocity”) decreased in all animals that were subjected to CPL (Figure 3e,f). These animals explored shorter distances of the arena with decreased speed, especially in the EarlySepsis group (Figure 3f). Surprisingly, sepsis does not impair exploration time; however, mice that suffered sepsis did move slower when freely exploring (Figure 3g). Although sepsis has been reported to impair short-term memory in rats [45], we were not able to detect any differences in the discrimination parameter D2 between the EarlySepsis and LateSepsis groups (Figure 3h). We did see a worsening in performance for the short-term discrimination index in the WT-Sepsis animals compared with Early and LateSepsis animals, but due to the high SD and low number of animals, there was no statistically significant difference (*p* > 0.05). Besides the low number of animals used to quantify behavior, one other limitation in the present study is the time difference between WT-Sepsis and LateSepsis groups, with the WT-Sepsis being analyzed after 3 months after the acute event, whereas the LateSepsis group had only 1 month to recover.

Due to the direct correlation between apoptotic neurons and an increase in microglia phagocytosis activity during the cellular phase of Alzheimer’s, we expected an increase in the cortical Iba1^+^ signal area of LateSepsis animals. However, we found a decreased iba1^+^ signal in the cortex of LateSepsis compared to EarlySepsis animals. This is even more surprising as the same animals also showed a decrease in the NeuN^+^ signal compared with the EarlySepsis group (Figure 4d,f, *p* = 0.016), clearly showing that neurodegeneration is taking place. Interestingly, this dynamic was not present at the hippocampal level. This could be an indication that sepsis may differently impact the cortex and hippocampus. It could also be a consequence of the difference in plaque formation between different brain structures in the animal model used. This is because in the APP mouse used, the formation of plaques occurs at around 3 months of age in the cortex and at around 4 months in the hippocampus [17,18]. We were able to confirm previous results showing a cortical astrocytic proliferation in LateSepsis animals (Figure 4e) consistent with the cellular response of the brain seen in AD. However, we could not also identify it at the hippocampal level, as previously reported [46]. Interestingly, we also could not identify any hippocampal cellular differences reported for the cortex (Figure 4d–g). This could be a consequence of either the type and low number of transgenic animals or the methods used to induce sepsis and measure its cortical and hippocampal outcome.

With increased evidence that the accumulation of Aβ in the brain of AD patients can be caused by low clearance rates, not necessarily a high production, several molecular and cellular mechanisms seem to contribute to amyloid removal [47]. One of the most interesting mechanisms is the “Glymphatic” system [48] in which the high expression of the AQP4 channel at the astrocyte end feet is thought to increase solute Aβ clearance [49,50]. As such, we also investigated the possibility that sepsis can directly affect the clearance of Aβ by disrupting AQP4 distribution. Interestingly, no difference in the distribution of AQP4 was seen around the large Sma^+^ vessel, but rather farther from it (Figure 4c,e), implying that parenchymal AQP4 is more impacted that AQP4 around large blood vessels. It is notable that LateSepsis animals displayed an increased AQP4 farther from the vessels and higher Aβ levels compared with EarlySepsis animals. This could be a consequence of an altered amyloid clearance in LateSepsis animals. None of the differences found for AQP4 distribution in the cortex were seen in the hippocampus, where an altered AQP4 distribution was not associated with an increase in Aβ levels. This might be due to regional differences in systemic sepsis.

## 5. Conclusions

As life expectancy increases, medical professionals will face more complex associations of pathologies, making the treatment more difficult to assign due to drug interactions and long-term effects of acute events. Establishing the cellular and molecular pathways affected in both acute and chronic conditions is essential for preventing and treating pathologies. Understanding how these mechanisms are affected in the presence of both conditions seems now more relevant to achieving a longer disability-free life expectancy.

## Data Availability

All data is available upon request.

## Supplementary Materials

The following supporting information can be downloaded at: https://www.mdpi.com/article/10.3390/cimb44090262/s1, Supplementary Table S1. Behavior results of APP animals that were subjected to sepsis. Supplementary Table S2. Signal areas measured in the cortex and hippocampus of APP mice that were subjected to sepsis. Supplementary Table S3. AQP4 signal area around SMA^+^ vessels quantified within a certain distance from the target.
