# Supplementary material for: Effects of Acute Sepsis on Cellular Dynamics and Amyloid Formation in a Mouse Model of Alzheimer’s Disease
Source: Curr Issues Mol Biol. 2022 Aug 24;44(9):3822–34. doi: 10.3390/cimb44090262 (PMC9497925; doi:10.3390/cimb44090262)
Supplement: Supplementary file 1 [file cimb-44-00262-s001.zip › cimb-1837343-supplementary.pdf]

Table S1. Behavior results of APP animals that were subjected to sepsis.

	Open Field Test												Novel Object Recognition Test			
	Speed (cm/s)				Distance (cm)				Time (s)				D2			
	Pre		5 Weeks		Pre		5 Weeks		Pre		5 Weeks		Pre		5 Weeks	
	Mean	SD	Mean	SD	Mean	SD	Mean	SD	Mean	SD	Mean	SD	Mean	SD	Mean	SD
**Early Sepsis**	5.73	0.72	2.87	0.87	3240.80	578.34	1512.14	534.04	160.75	27.48	110.63	87.46	0.31	0.09	0.17	0.17
**Late Sepsis**	4.26	0.83	3.20	1.64	3101.23	330.75	2039.58	951.10	179.85	51.13	98.57	49.25	0.37	0.29	0.31	0.48
**APP Sham**	5.70	1.32	7.27	1.11	3325.93	782.28	3048.52	706.48	152.86	29.59	88.79	36.56	0.47	0.21	0.44	0.22
**WT Sepsis**	5.29	0.73	2.24	0.39	2944.35	459.23	1241.17	228.81	151.11	30.08	67.01	40.14	0.52	0.15	0.05	0.36
**WT-Sham**	5.01	1.02	4.88	0.72	2759.25	386.94	2860.51	582.90	162.04	36.14	137.11	26.52	0.54	0.15	0.465	0.12

Table S2. Signal areas measured in the cortex and hippocampus of APP mice that were subjected to sepsis.

		NeuN (%)		GFAP (%)		Iba1 (%)		Aβ (%)	
		Mean	SD	Mean	SD	Mean	SD	Mean	SD
**Cortex**	**Early Sepsis**	7.04	1.73	1.94	1.11	1.22	0.80	0.36	0.17
	**Late Sepsis**	4.09	2.11	4.84	3.10	0.28	0.20	0.71	0.37
	**APP Sham**	6.69	1.25	2.77	0.47	1.31	0.15	0.29	0.23
	**WT Sepsis**	6.80	2.16	1.39	1.60	0.72	0.25	0	0
	**WT-Sham**	8.14	1.19	2.35	0.53	0.97	0.15	0	0
**Hippocampus**	**Early Sepsis**	5.22	1.20	2.87	1.11	1.02	0.90	0.12	0.09
	**Late Sepsis**	3.72	2.30	5.36	2.10	0.44	0.45	0.24	0.12
	**APP Sham**	3.80	0.43	3.60	0.58	1.06	0.54	0.28	0.13
	**WT Sepsis**	3.91	1.91	3.02	2.68	0.29	0.13	0	0
	**WT-Sham**	4.67	0.72	3.16	0.53	0.66	0.22	0	0

Table S3. AQP4 signal area around SMA^{+} vessels quantified within a certain distance from the target.

	Cortex					Hippocampus				
	50 µm	100 µm	150 µm	200 µm	250 µm	50 µm	100 µm	150 µm	200 µm	250 µm
APP-Sham vs. WT-Sepsis - #	0,8159	0,1191	<0,0001	0,0002	<0,0001	0,7495	0,9868	>0,9999	0,6216	0,1497
APP-Sham vs. EarlySepsis - $	0,8545	0,5295	<0,0001	0,0008	<0,0001	0,9496	0,7678	0,9748	0,9762	0,9675
APP-Sham vs. LateSepsis - &	0,8732	0,7226	0,6476	0,7484	0,7146	0,8324	0,5988	0,4178	0,2341	0,0474
WT-Sepsis vs. EarlySepsis - *	0,9993	0,7412	0,3303	0,9234	0,6684	0,9547	0,5518	0,9796	0,3366	0,04
WT-Sepsis vs. LateSepsis - ‡	0,9969	0,0041	<0,0001	0,0015	<0,0001	0,9944	0,3762	0,3997	0,938	0,9902
EarlySepsis vs. LateSepsis - ©	0,9998	0,0509	0,001	0,0061	0,0002	0,99	0,994	0,1593	0,0699	0,0072
